# Visual properties of human retinal ganglion cells

**DOI:** 10.1371/journal.pone.0246952

**Published:** 2021-02-16

**Authors:** Katja Reinhard, Thomas A. Münch

**Affiliations:** 1 Retinal Circuits and Optogenetics, Centre for Integrative Neuroscience and Bernstein Center for Computational Neuroscience, University of Tübingen, Tübingen, Germany; 2 Neuroscience Graduate School, University of Tübingen, Tübingen, Germany; 3 Institute for Ophthalmic Research, University of Tübingen, Tübingen, Germany; Doheny Eye Institute/UCLA, UNITED STATES

## Abstract

The retinal output is the sole source of visual information for the brain. Studies in non-primate mammals estimate that this information is carried by several dozens of retinal ganglion cell types, each informing the brain about different aspects of a visual scene. Even though morphological studies of primate retina suggest a similar diversity of ganglion cell types, research has focused on the function of only a few cell types. In human retina, recordings from individual cells are anecdotal or focus on a small subset of identified types. Here, we present the first systematic *ex-vivo* recording of light responses from 342 ganglion cells in human retinas obtained from donors. We find a great variety in the human retinal output in terms of preferences for positive or negative contrast, spatio-temporal frequency encoding, contrast sensitivity, and speed tuning. Some human ganglion cells showed similar response behavior as known cell types in other primate retinas, while we also recorded light responses that have not been described previously. This first extensive description of the human retinal output should facilitate interpretation of primate data and comparison to other mammalian species, and it lays the basis for the use of *ex-vivo* human retina for in-vitro analysis of novel treatment approaches.

## Introduction

Vision starts in the retina, a highly structured part of the central nervous system. The retina performs important signal processing: the incoming visual images are captured by the photoreceptors, analyzed and split into parallel information streams by retinal circuits, and sent along the optic nerve to higher visual brain centers. Each of the parallel information streams is embodied by a type of ganglion cell and informs the brain about a particular aspect of the visual scene [1]. The non-primate mammalian retina contains over 40 of these different information streams, which can be distinguished based on functional, morphological, and genetic criteria [2–7].

One striking aspect of retinal architecture is that each ganglion cell type tiles the retina so that each visual feature can be extracted at each location in the visual field. Nevertheless, regional specializations do exist, for example the fovea of the primate retina, a region of very high visual acuity. The foveal region consists almost exclusively of four retinal ganglion cell types, the ON and OFF parasol cells, and the ON and OFF midget cells [8–10], which account for 50–70% of all ganglion cells in the primate retina [11, 12]. Functional studies using human and non-human primates have often focused on these four most abundant retinal ganglion cell types [13–20]. Morphological and transcriptomic studies of the complete primate retina agree that midget and parasol cells are the dominating cell types, but these studies describe a similar variety in the remaining ganglion cell types as found in the non-primate retina with at least 12 additional types [11, 12, 21–24]. However, functional studies of these non-foveal ganglion cell types in non-human primates have been limited to a set of 7 types [15, 25–30] and only midget and parasol cells have been characterized in human retina [19, 20]. Additional physiological assessment of the human retina on the level of individual cells is anecdotal [31, 32].

In this study, we present a survey of ganglion cell function in the non-foveal human retina. We performed multi-electrode array (MEA) recordings on *ex-vivo* retinas obtained from enucleation patients and recorded light-driven activity from dozens of human ganglion cells in parallel. MEAs have been successfully used in previous studies to characterize the retinal output in various animal models [4, 13, 33–38]. Our data represents the first systematic and non-selective recording and characterization of light responses from a large population of ganglion cells in human retina. In addition to providing an overview of the spectrum of light responses in the human retina, we compare the representation of the spatio-temporal stimulus space by human ganglion cells with published data from non-human primate retina and results from psychophysical studies.

## Results

### Recording light responses from donated human retinas

To record light responses from human retinal ganglion cells, we obtained human retinas from patients who had to undergo enucleation of one eye due to a uveal tumor. Retinal pieces (~ 3 x 3 mm^2^) were placed ganglion cell-side down onto multi-electrode arrays and responses to a set of light stimuli were recorded at photopic light intensities. Individual stimuli (gray-scale images) spanned at most 3 log units of brightness. Spikes were assigned to individual units (presumably retinal ganglion cells) during an offline, semi-manual spike sorting process based on principal component analysis of spike waveforms. Only clearly sortable units were considered for analysis (see Method section for details). In total, we obtained the spiking activity of 342 light-responsive single units in 15 retinal pieces obtained from 10 human retinas (Table 1).

**Table 1:** Human retinas used for this study.

**Donor**				**Surgery conditions**		**Retina**	**Experiment notes**			**Light responses**				
**ID**	Sex	Age	Notes	Ischemia (min)	Dark lens	Retinal part (ventr./dors./temp./nasal)	Preparation	# Analyzed pieces	Any light resp.?	DG	Chirp	Bar	Flash	#
**1**	m	72	diabetic	7	-	Vt	easy	1	yes	18	-	2	15	19
**2**	f	49	***radiation***	7	-	Dn		-	-					
**3**	f	72	diabetic	7	-	Dn	rolling	1	yes	29	-	4	15	29
**4**	f	69	***detachment***	***17***	-	N	easy	1	yes					
**5**	m	53		7	-	T	sticky	1	yes	15	9	0	1	20
*6	m	75	sinus tumor	***18***	-			-	-					
**7**	m	89		***25***	-	Dn	sticky	-	yes					
**8**	f	42		***20***	-	Vt	sticky	-	-					
**9**	f	83		10	-	T	sticky	1	yes	22	21	9	8	29
**10**	f	49		10	-	T	sticky	3	yes	54	10	1	3	60
**11**	f	60		7	yes	Vn	sticky	3	yes	86	68	11	42	88
**12**	f	74	macular edema	7	yes	Dt	easy	2	yes	23	15	0	26	35
**13**	m	74		7	yes	V	***very sticky***	-	yes					
**14**	f	79	radiation 10y ago	8	-	N	easy	1	yes	13	12	10	8	16
**15**	m	67	detachment	10	-	N	easy	1	yes	29	6	0	3	30

15 *ex-vivo* human retinas were obtained. The table contains information about the donor (sex, age, known medical history), surgery conditions (the ischemia duration, i.e. time without oxygen and nutrient supply, and whether a dark lens was put on the donor’s eye during surgery), and the retina (left/right eye, part of the retina without tumor). During preparation, the retina would sometimes roll up immediately after vitrectomy (rolling) or the vitreous was sticking strongly to the retina (sticky). The following two columns indicate how many retinal pieces were used per retina for the final analysis and whether any light responses were detected in our recordings. The last section indicates the number of cells responding to four types of stimuli (DG: drifting-grating); the last column shows the number of cells responding to any of the four stimuli. Light gray rows: retinas with few light responses, not used for analysis. Dark gray rows: no detectable light responses. Bold: potential reasons for low quality. Ventr./v = ventral, dors./d = dorsal, temp./t = temporal, n = nasal, m = male, f = female, radiation = radiation of the tumor-bearing eye, detachment = partial retinal detachment prior to surgery, resp. = responses. *retina prepared by another group during a different study.

### Response properties across the population of ganglion cells

**Fig 1:**
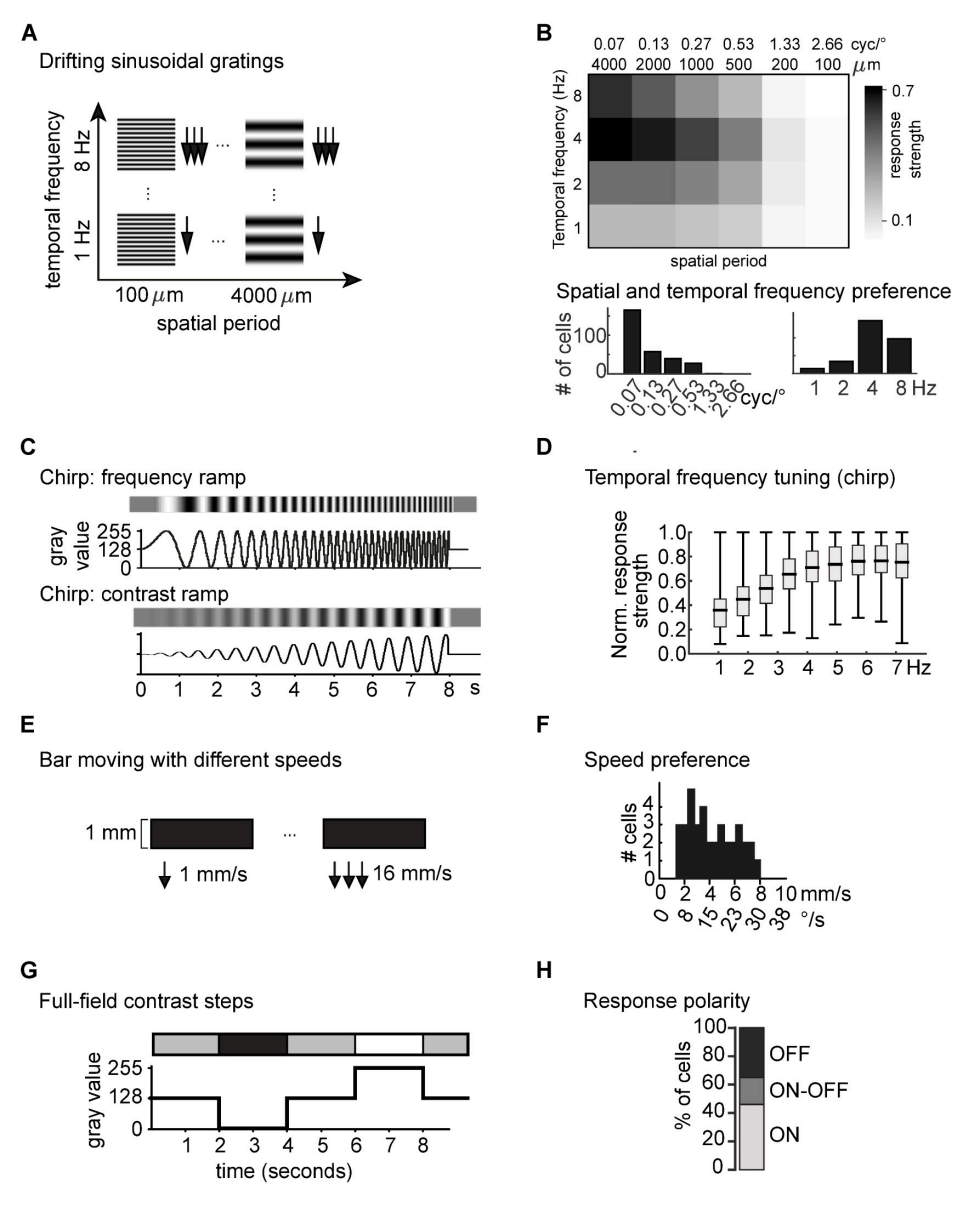
Population response of human retinal ganglion cells. (A) Drifting gratings with 4 temporal frequencies and 6 spatial frequencies. (B) Response strength (amplitude of the Fourier transform (FT) at the stimulus frequency, normalized to maximal response) to 24 drifting sinusoidal gratings with different spatial and temporal frequencies, averaged across N = 293 cells (top). Distribution of preferred spatial (left) and temporal (right) frequencies in response to drifting gratings, N = 288 cells (bottom). (C) Full-field “chirp” frequency ramp from 0.5 to 8 Hz and full-field “chirp” contrast ramp. (D) Normalized response strength (FT_response_/FT_stimulus_) to a full-field frequency ramp (“chirp”), in different frequency bands (Box-whisker-plots: mean, quartiles, maximum and minimum; N = 141 cells). (E) Bar moving with 6 different speeds. (F) Distribution of the median preferred speed measured with a single moving bar, N = 37 cells. (G) Full-field flash contrast steps consisting of two positive and two negative contrast steps. (H) Proportion of ganglion cells responding to positive full-field contrast steps (ON), negative contrast steps (OFF) or both (ON-OFF), N = 121 cells.

We aimed at characterizing the diversity of the output of the human retina with different visual stimuli (Fig 1). We will first give an overview of the stimuli and the ganglion cell responses at the population level. We used drifting-grating stimuli to characterize the encoding of the spatio-temporal space (Fig 1A). Of the 342 light-responsive cells, 86% responded to these stimuli. As a population, the recorded cells responded to a large spatio-temporal stimulus space including all tested spatial frequencies (100 to 4000 μm spatial period on the retina, corresponding to 2.66 to 0.07 cycles per degree (cyc/°)) and temporal frequencies (1 to 8 Hz) with an overall preference for stimuli of 500 to 4000 μm retinal size (0.53 to 0.07 cyc/°) and moving with 2 to 8 Hz (Fig 1B). Fig 1B (top) shows the response strength averaged across all recorded cells to the 24 different sinusoidal drifting gratings. To obtain the displayed heat-map, the amplitude of the Fourier transform of the cells’ responses at the stimulus frequency was taken as response strength and normalized for each cell across the 24 grating stimuli. The distribution of preferred spatial and temporal frequencies per cell are shown in Fig 1B (bottom; maximum out of the 24 drifting-grating combinations). While the recorded ganglion cells showed responses to a broad range of spatial and temporal frequencies (Fig 1B top), they mostly responded best to coarse gratings (Fig 1B bottom left) and higher temporal frequencies (Fig 1B bottom right).

Temporal frequency preferences were further measured with a full-field frequency ramp (“chirp” stimulus, Fig 1C top) which has proven to be an excellent stimulus to classify the behavior of retinal ganglion cells [2]. The chirp stimulus was only used in 12 out of the 15 retinas and drove activity in 51% of our analyzed cells from these 12 retinas. Here, response strength was defined as the ratio of the Fourier transform of the cells’ response and the Fourier transform of the stimulus. We analyzed this normalized response strength across all cells with chirp responses in discrete 1 Hz bins. Fig 1D shows the distribution of the normalized response strength for each bin across all responding cells (mean, quartiles and extremes). While there was at least one cell with a maximum response for each frequency bin, this chirp stimulus confirmed a general preference of the human retinal output for higher temporal frequencies. We also presented a chirp stimulus with a contrast ramp (Fig 1C, bottom), ganglion cell responses to this stimulus are shown further down.

Bars moving with different velocity (Fig 1E) were used to test speed preference of ganglion cells and elicited clear responses in 11% of all cells. The distribution of the median preferred speeds (50% of the cumulative sum of the response amplitudes) was rather wide, ranging from bars moving between 2 and 8 mm/s retinal speed (7.5 to 30°/s) in different ganglion cells (Fig 1F).

Finally, response polarity was tested with full-field contrast steps (Fig 1G). Over a third of the recorded cells responded consistently to this stimulus. Of those cells, 46% responded solely to positive full-field contrast-steps (ON-responses), 35% showed responses to negative contrast steps (OFF-responses), and the remaining 19% responded to both (ON-OFF-responses; Fig 1H).

### Diversity in the output of human retina

**Fig 2:**
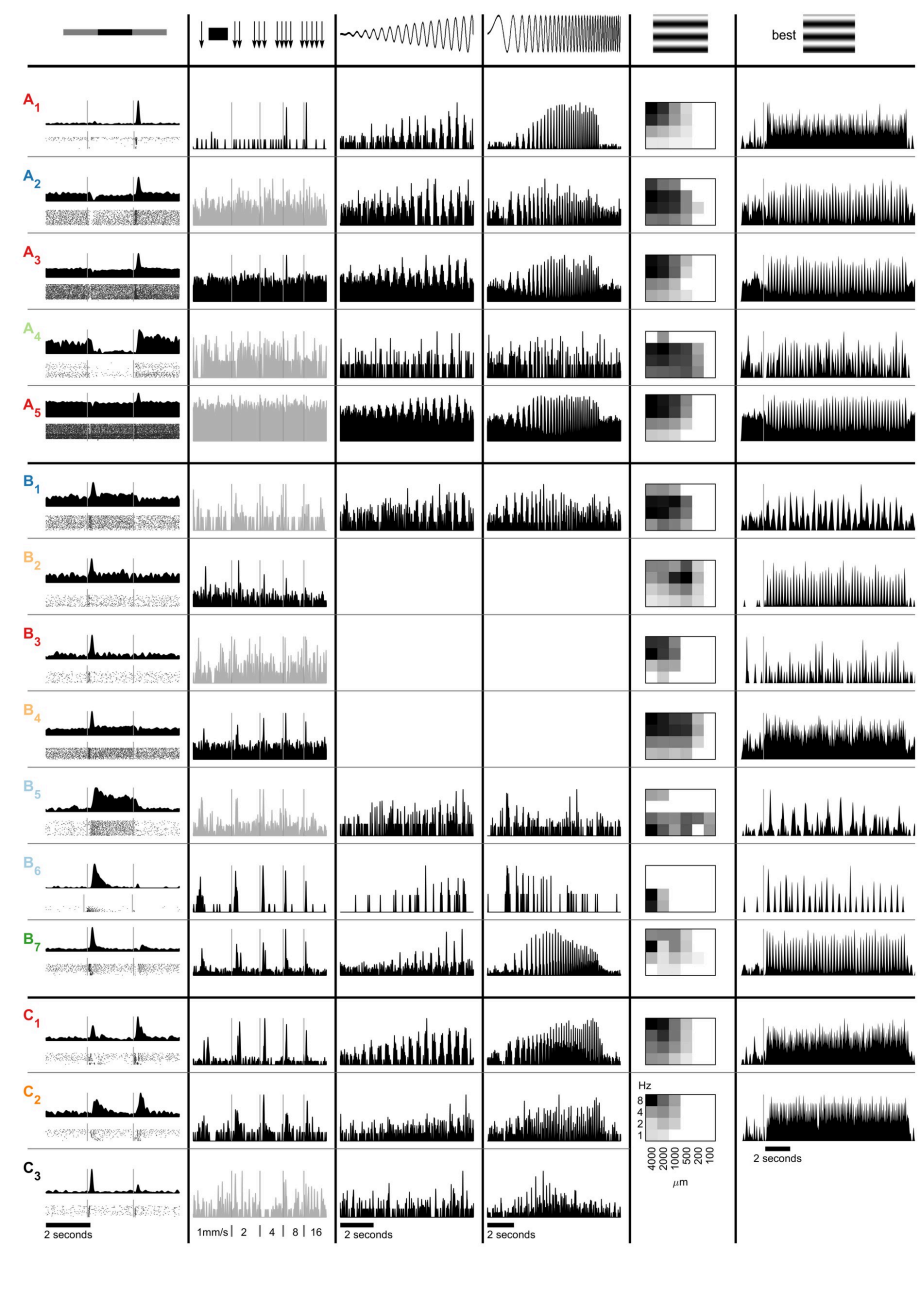
Example response properties of human ganglion cells. A) ON-cells, B) OFF-cells, C) ON-OFF-cells. Column 1: average firing rates (top) and raster plots (bottom) to full-field contrast steps; column 2: response to bar moving with different speeds; column 3: activity during a full-field chirp contrast ramp at 2 Hz; column 4: response to “chirp stimulus” (full-field temporal frequency modulation from 0.5 to 8 Hz); column 5: normalized response strengths to 24 sinusoidal drifting-gratings (convention as in Fig 1B); column 6: firing rate for the sinusoidal drifting-grating causing maximal response (black square in column 5). Stimuli are depicted on top. Colors correspond to Fig 5. Empty space indicates that the stimulus was not presented to this cell. Firing rates plotted in gray indicate that this cell did not respond consistently to this stimulus.

One hallmark of the retina is the separation of visual information into different information streams embodied by distinct ganglion cell types. When analyzing individual cells, we found a wide range of response properties to our set of light stimuli, illustrated with 15 example cells in Fig 2. These example cells span the range of observed response polarity and transiency, spatio-temporal preferences, contrast sensitivity, and responsivity to local stimuli. While the description of those 15 cells is necessarily anecdotal in nature, it provides a good intuition for the sometimes subtle, sometimes substantial differences in response properties. Later, we will provide a more rigorous, cluster-based grouping of cells. For the following description, we group these 15 cells based on their responses to full-field contrast steps (column 1 in Fig 2), with cells responding only to positive contrast steps (ON-cells) in Fig 2A_1_–2A_5_, cells responding exclusively to negative contrast steps (OFF-cells) in Fig 2B_1_–2B_7_, and cells responding to both (ON-OFF cells) in Fig 2C_1_–2C_3_.

#### ON-cells

The cell A_1_ in Fig 2 showed a very transient response to a positive contrast step (at the transition from black to gray full-field stimulation, column 1). It preferred coarse drifting-gratings with a high temporal frequency (columns 5 and 6), which is consistent with the steep increase in responsivity for a spatially homogeneous frequency ramp (column 4). The cell also responded to stimulation with a spatially less homogenous stimulus, a fast moving bar (column 2). When probed with a contrast ramp, the activity of this cell was already modulated at relatively low contrast (column 3). A_2_ and A_3_ are additional transient ON-cells. Compared to the first cell, these cells responded well to a broader spectrum of temporal frequencies (columns 4 and 5), but cell A_2_ did not show significant activity modulation in response to a moving bar (column 2).

Some recorded ganglion cells had very high spontaneous firing rates such as the cells A_4_ and A_5_ (column 1). Nevertheless, they precisely encoded various combinations of temporal and spatial frequency stimuli and showed selective activity modulations to their preferred contrast step. In addition, some cells were strongly inhibited by negative contrast, like example cell A_4_ (column 1).

#### OFF-cells

Four examples of transient OFF-cells are shown in Fig 2B_1-4_. Only cells B_2_ and B_4_ responded consistently to moving bars, but with opposite preferences (B_2_ preferred slow bars, B_4_ fast bars, column 2). Their speed preferences are also reflected in their responses to drifting gratings (column 5). Both cells preferred similar temporal frequencies (4–8 Hz), but different spatial frequencies resulting in distinct speed preferences (1–2 mm/s for B_2_ and 16–32 mm/s for B_4_). B_3_ responded only to fast and wide stimuli and not to moving bars, while B_1_ responded to a broad range of spatial frequencies and slower stimuli (column 5).

The sustained cells B_5_ and B_6_ both preferred low temporal frequencies (1–2 Hz) when probed with a drifting-grating stimulus (column 5) or the chirp stimulus (column 4). The less sustained cell B_6_ responded strongly to all moving bars (column 2), while the cell B_5_ did not respond consistently to this stimulus. Finally, the OFF-cell B_7_ exhibited a rebound or delayed response to positive contrast after an initial inhibition (column 1). The cell showed a preference for temporal frequencies around 4 Hz (column 5) and higher contrast stimuli (column 3), and it responded well to all speeds of a moving bar (column 2).

#### ON-OFF-cells

ON-OFF cells may show rather sustained (cells C_1_ and C_2_) or transient responses (C_3_, column 1). Some clearly preferred higher temporal frequencies (C_1_), others responded only to low frequencies (C_3_, column 4). While the cell C_1_ responded well to the whole contrast ramp, the other two example cells showed some activity modulation only to maximal contrast (column 3). Interestingly, cell C_3_ responded well to temporal frequencies around 3 Hz when exposed to full-field stimulation (chirp stimulus, column 4) but did not respond to moving bars (column 2).

### Spatio-temporal properties of human ganglion cells correspond to psychophysical detection threshold

**Fig 3:**
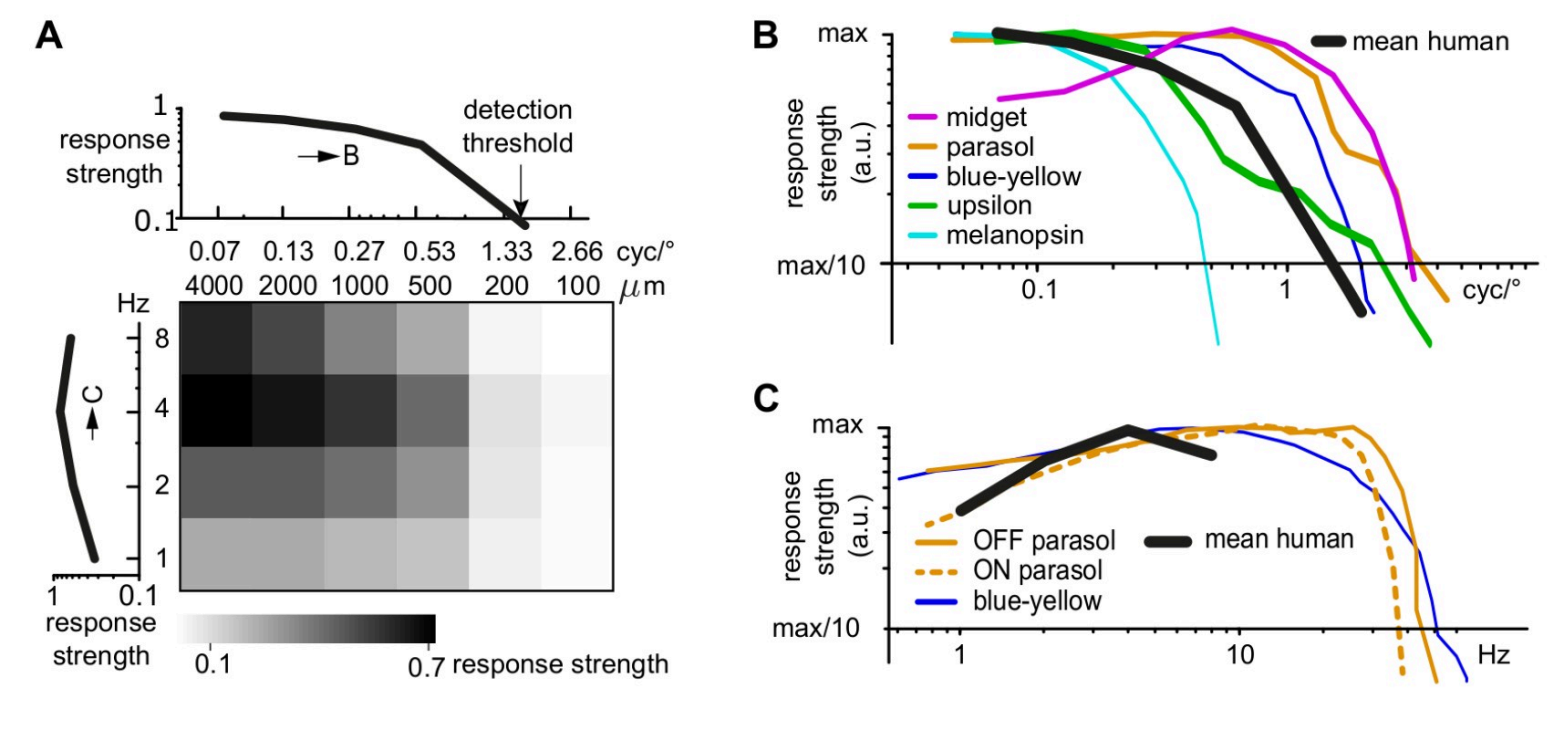
Human retinal ganglion cells show similar spatial and temporal frequency response curves as non-human primate retinal ganglion cells. (A) Heat map: Average responsivity of human retinal ganglion cells for drifting sinusoidal gratings, replicated from Fig 1E (N = 293). Curves: Spatial frequency (top) and temporal frequency (left) response curves (mean across all cells). (B, C) Spatial and temporal response curve in comparison with published data on non-human primate ganglion cells. Non-human primate data adapted from [40] (midget); [41, 42] (parasol); [41, 43] (blue-yellow); [25] (upsilon); [28] (melanopsin).

The responses to drifting or sign-inverting grating stimuli have often been used to characterize, identify, and compare different retinal ganglion cell types. We therefore explored in more detail how the spatio-temporal stimulus space is encoded by the human retina. The heat-map in Fig 1E (replicated in Fig 3A) indicates along the vertical axis that the mid-peripheral human retina responds well to all presented temporal frequencies, with an optimum at 4 Hz, and shows a general preference for coarser stimuli (horizontal axis of the heat map). To directly compare the human retina responses to published human psychophysics data, we computed the spatial response curve of the whole population of recorded cells (Fig 3A top and 3B). This curve was achieved as follows: for each cell, we first determined the optimal temporal frequency, and plotted a normalized spatial frequency response curve at this optimal temporal frequency. Then we averaged these individual spatial response curves. In the corresponding way, the average temporal response curve was calculated (Fig 3A left and 3C). The average spatial response curve dropped below 10% of its maximum for stimuli of 1.55 cyc/° and finer (Fig 3A top). This *in-vitro* spatial threshold corresponds well to previously determined psychophysical detection thresholds in the mid-peripheral visual field (4 cyc/° at 14° visual angle and 2 cyc/° at 30°) [39].

### Comparison to existing human and non-human primate data

Temporal and spatial frequency preferences have been used as the main parameter in several studies on non-human primate retina to characterize and identify different ganglion cell types [25, 28, 40–43]. In all non-human primate publications considered here for comparison with our human ganglion cell data, response strength has been given either as absolute number of spikes or as a normalized amplitude of the Fourier transform of the cells’ responses. We extracted the response curves from these publications (midget ganglion cells [40]; parasol ganglion cells [41, 44]; blue-yellow ganglion cells [41, 43]; upsilon ganglion cells [25]; melanopsin ganglion cells [28]) and overlaid them with the population tuning curves obtained from our human ganglion cell data, as shown in Fig 3B and 3C. Both the average spatial response curve (Fig 3B) and the average temporal response curve (Fig 3C) for the human retina lie within the range of published data from different primate ganglion cell types.

Proper classification of the recorded cells would require morphological information and/or denser electrophysiological recordings to reveal mosaic formation. However, it is well established that the most common ganglion cell types in the primate retina are midget and parasol cells [9–11]. Midget cells respond to a broad range of spatial frequencies, show sustained responses and low contrast gain [9]. Parasol cells, on the other hand, have transient responses, respond well to many spatial frequencies, but especially to wide stimuli, show strong responses to high temporal frequencies, and exhibit a high contrast gain [9, 41]. To get a more systematic handle on the response diversity in our data, as well as the representation of the known major primate ganglion cell types, we compared our data to the reported response properties in Cowan et al. 2020 [45] which used the same ‘chirp’ stimulus as we did. Cowan and colleagues grouped their recorded flash and chirp responses into five clusters. Based on the overrepresentation of parasol, midget and ON-OFF bistratified cells in the human retina, we can assume that their ON and OFF sustained clusters are dominated by ON and OFF midget cells, the ON and OFF transient clusters by ON and OFF parasol cells, and the ON-OFF cluster by ON-OFF bistratified cells.

**Fig 4:**
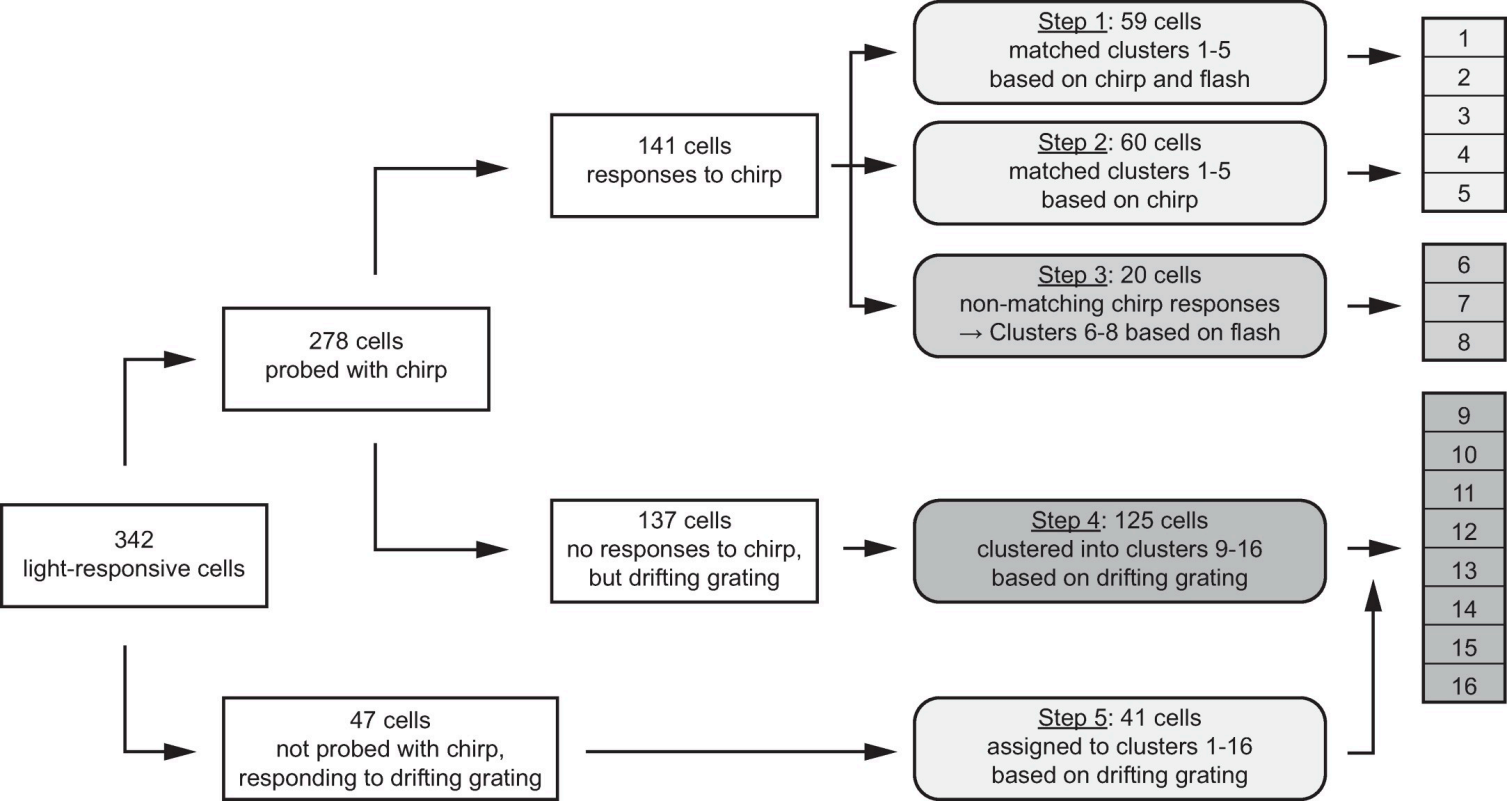
Schematic of clustering process. Cell responses were clustered in a 5-step process. Clustering was focused on cells that were probed with the chirp stimulus. All cells with response to the chirp were assigned to the 5 Cowan clusters if their responses matched (step 1 and 2, cluster 1–5), otherwise they were grouped based on their flash responses (step 3, clusters 6–8). Cells without chirp responses were clustered based on their drifting-grating responses (step 4, clusters 9–16). Cells that were not probed with the chirp stimulus were assigned to one of the 16 clusters based on their drifting-grating responses (step 5). At each step, a few cells dropped out if they did not fit the parameters of the current nor the next step. E.g. cells that did not experience the chirp stimulus and did not respond to the drifting-grating stimulus did not enter the 5 step process (17 cells).

**Fig 5:**
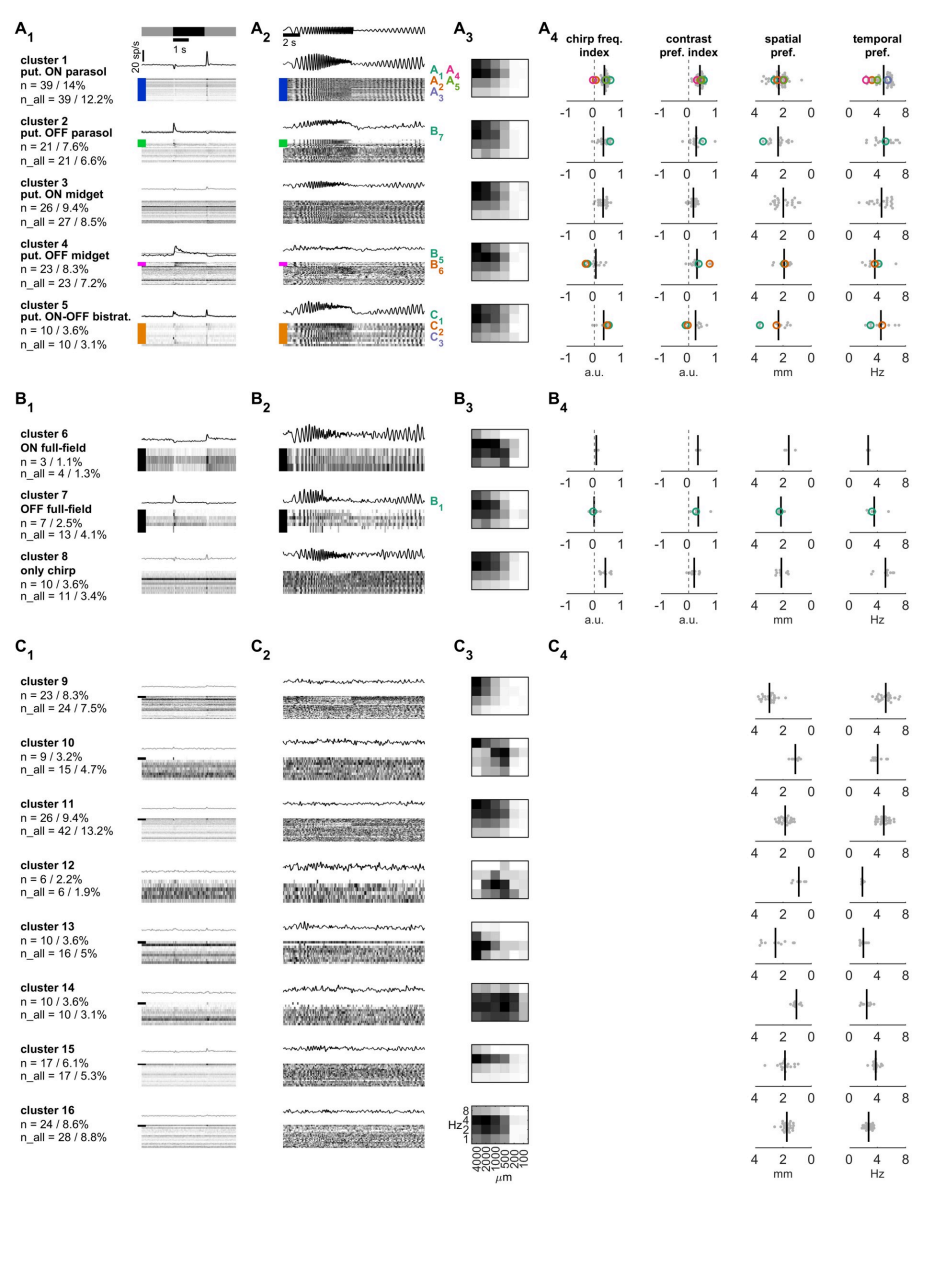
Clusters of human retinal ganglion cells. (A) Clusters of cells that putatively correspond to the five dominant primate retinal ganglion cell types, assigned based on their chirp and flash responses. (B) Clusters with chirp responses that do not fit cluster 1–5. Cells were clustered based on their flash responses. (C) Clusters of cells without chirp responses were clustered based on their drifting-grating responses. The remaining cells that were not probed with the chirp stimulus were assigned to the previous 16 clusters based on their drifting-grating responses. (A_1_, B_1_, C_1_) Left: Name of each cluster, number n and percentage of cells relative to total of light-sensitive cells that were probed with the chirp stimulus. n_all includes cells that were not probed with the chirp stimulus but assigned based on their drifting-grating responses. Right: Heatmap of responses to the flash stimulus of all cells in each cluster (spontaneous background firing rate subtracted; horizontal axis is time; each line is one cell). Colored bars here and in A_2_ indicate the cells with a flash response, the color corresponds to the scheme used in Cowan et al. 2020 [45]. Above the heatmap, the cluster average is shown in black for cells with flash responses and in gray for the remaining cells. (A_2_, B_2_, C_2_) Normalized responses of all cells in each cluster to the chirp stimulus (heatmap, 1 line per cell, black indicates max. response). Numbers on the right refer to the example cells in Fig 2. (A_3_, B_3_, C_3_) Average, normalized response to the set of 24 drifting-grating stimuli for all cells in each cluster. (A_4_, B_4_, C_4_) Distribution of four indices for each cell (gray) in the cluster. Example cells form Fig 2 are indicated in color. Solid lines indicate median values. Chirp frequency index: <0 indicates preference for lower temporal frequencies in the chirp stimulus, >0 indicates a preference for higher temporal frequencies. Contrast preference index: <0 indicates a preference for the lower contrast part of the chirp stimulus, >0 a preference for higher contrasts. Spatial preference and temporal preference: the values indicate the location of the peak of a Gaussian fit to the drifting-grating heatmap.

To compare our data to Cowan et al. 2020, we initially only considered cells in our data set that were probed with the chirp stimulus and responded to at least one of the shown light stimuli (n = 278). The assignment and clustering process is illustrated in Fig 4 (details in Methods). We first assigned cells to the five Cowan clusters based on the similarity to their flash and chirp responses (Figs 4, 5A). 59 cells were assigned to the five Cowan clusters based on the similarity of their chirp- and flash-responses (Step 1 in Fig 4), another 60 cells based only on their chirp responses (Step 2), for a total of 119 out of the 278 cells (43% of chirp-probed cells: 14% putative ON parasol cells, 7.6% putative OFF parasol cells; 9.4% putative ON midget cells, 8.3% putative OFF midget cells; 3.6% putative ON-OFF bistratified cells; Fig 5A Clusters 1–5). Next (Step 3 in Fig 4), cells that responded to the chirp stimulus, but with a different response profile than the Cowan clusters, were grouped based on their flash responses into 3 more clusters: transient ON (Fig 5B Cluster 6), transient OFF (Fig 5B Cluster 7) and without flash responses (Fig 5B Cluster 8). Finally, most of the cells that did not show any responses to the chirp stimulus showed responses to the set of drifting-grating stimuli (125 out of 137 cells) and were hence clustered based on their peak drifting-grating responses using k-means (Step 4 in Fig 4). This resulted in additional 8 clusters (Fig 5C, Cluster 9–16). Last, the remaining 47 cells not probed with the chirp stimulus were assigned to these 16 clusters based on their responses to the drifting gratings (Step 5 in Fig 4).

To describe and quantify the response behavior of cells within each cluster, we quantified the responses to the chirp stimulus and drifting-gratings with four parameters (Fig 5A_4_, 5B_4_ and 5C_4_; see Methods for more details): The chirp frequency index is based on the Fourier transform of the responses to the frequency ramp. The Fourier transform of the cell response was normalized and divided by the Fourier transform of the stimulus itself. The index indicates the preference of the cell for higher (>0) or lower (<0) temporal frequencies. The contrast preference index was calculated based on the absolute amplitude of the background-subtracted cell response to the chirp contrast ramp. Cells that prefer low contrast have an index of <0, high-contrast preferring cells an index of >0. As an additional measure of frequency preferences of human ganglion cells, we fit a Gaussian to the heatmaps of the responses to drifting-gratings (see Methods). The spatial and temporal preference in column 3 and 4 of Fig 5A_4_, 5B_4_ and 5C_4_ represent the peaks of these Gaussians along the spatial and temporal axis. Averages and standard deviations for each cluster and parameter are summarized in Table 2.

**Table 2:** Response behavior of clustered human retinal ganglion cells.

Cluster	N	Chirp frequency index [a.u.]	Contrast preference index [a.u.]	Spatial frequency preference [μm]	Temporal frequency preference [Hz]
**1**	39	-0.36 ± 0.15	0.4 ± 0.12	1704 ± 547	4.9 ± 1.2
**2**	21	-0.32 ± 0.18	0.27 ± 0.24	1652 ± 616	4.9 ± 1.47
**3**	26	-0.31 ± 0.16	0.17 ± 0.12	2019 ± 655	4.6 ± 1.36
**4**	23	-0.05 ± 0.22	0.29 ± 0.26	2078 ± 425	3.6 ± 1.22
**5**	10	-0.33 ± 0.21	0.24 ± 0.29	1678 ± 814	4.5 ± 1.62
**6**	3	-0.07 ± 0.06	0.33 ± 0.07	2407 ± 407	2.7 ± 0.15
**7**	7	0.01 ± 0.16	0.34 ± 0.26	1854 ± 168	3.6 ± 1.21
**8**	10	-0.39 ± 0.16	0.2 ± 0.16	1883 ± 352	5.1 ± 1.13
**9**	23	no data	no data	1870 ± 1406	5.2 ± 1.05
**10**	9	no data	no data	3353 ± 133	5.2 ± 1.05
**11**	26	no data	no data	2153 ± 390	4.9 ± 0.77
**12**	6	no data	no data	3116 ± 415	1.9 ± 0.15
**13**	10	no data	no data	1467 ± 928	2 ± 0.43
**14**	10	no data	no data	2948 ± 258	2.5 ± 0.66
**15**	17	no data	no data	2138 ± 537	3.8 ± 0.48
**16**	24	no data	no data	2270 ± 322	2.8 ± 0.51

Mean and standard deviation of the parameters depicted in Fig 5. Chirp frequency and contrast preference index were calculated based on responses to the chirp stimulus. A frequency index >0 indicates preference for higher temporal frequencies, an index <0 preference for lower frequencies. A contrast preference index <0 indicates a preference for lower contrast, an index >0 preference for higher contrast. Spatial and temporal frequency preferences were defined as the peak of a Gaussian fitted to the heatmap of responses to 24 drifting-gratings.

The putative parasol cells in Clusters 1 and 2 that did respond well to full-field stimulation showed the typical transient response profile (Fig 5A_1_) and strong responses across all temporal frequencies tested with the chirp stimulus (Fig 5A_2_). As expected from literature [9, 41], they showed a broad response profile to the different spatial frequencies and tend to prefer higher temporal frequencies (Fig 5A_3_ and 5A_4_). All our example ON cells in Fig 2 (A_1-5_) were assigned to Cluster 1, despite their more subtle individual differences described above, while example cell B_7_ is a putative OFF parasol cell (Cluster 2). The putative midget cells in Cluster 3 and 4 were assigned based on the very consistent chirp responses across all temporal frequencies as described for the sustained ON and OFF clusters in Cowan et al. 2020 [45]. Similar to the cells in Clusters 1 and 2, the putative midget cells responded to a broad range of spatial frequencies. Our example cells in Fig 2 contain two putative OFF midget cells (B_5_ and B_6_). Consistent with the reported lower contrast gain in midget cells [9], we found a contrast preference index closer to 0 in putative ON midget cells. In putative OFF midget cells, the contrast responses to full-field chirp were generally weaker and more variable. Finally, the putative ON-OFF bistratified cells in Cluster 5 showed strong responses to high temporal frequencies and a broad response profile to spatial frequencies.

The chirp responses of the cells in Clusters 6–8 (Fig 5B) differed from the responses of the five dominant types. We clustered them based on their full-field responses. Cells in Cluster 6 had ON responses, and cells in Cluster 7 OFF responses, both were sensitive to temporal frequencies predominantly in the low to medium range. Cells in cluster 8 did not respond consistently to the full-field flashes, but they responded well to medium to higher temporal frequencies.

Finally, the cells in Clusters 9–16 did not respond to the chirp stimulus. We clustered them based on their responses to drifting-gratings. There is only a very small subset that responded to a full-field flash (black bars in Fig 5C_1_), but in general, these clusters expressed specific spatio-temporal tunings. For example, cells in Cluster 9, 12, 13 and 15 had very narrow and distinct tuning especially in their temporal responses. Cells in Cluster 14 responded to a very broad set of temporal and spatial frequencies, but nevertheless had a consistent peak at low temporal frequencies and for narrow stimuli. Further, cells in Clusters 10 and 12 had a very specific response behavior with a diagonal pattern of spatio-temporal preferences, suggesting a potential speed-encoding function.

Taken together, almost half of the cells in our data set can be assigned to the dominant five types of ganglion cells in the primate retina, while the remaining cells can be grouped into 11 additional clusters resulting in a total of 16 putative ganglion cell types.

### *Ex-vivo* human retinas show physiological properties of healthy tissue

One potential problem when working with human retinas is the unclear health status of the donor tissue. We obtained retinas from donors between 42 and 89 years of age and with different medical histories (Table 1). In addition to the variability introduced by the donors, several circumstances can harm the tissue and prevent light responses: Depending on the surgery procedure, the retina of the ligated eye bulb might have been exposed to longer periods without oxygen and nutrients (ischemia). Furthermore, because of the growing tumor, the retina might have been detached from the pigment epithelium prior to the surgery, which is particularly harmful to photoreceptors. In this study, we thus excluded all retinas exposed to >10 min of ischemia (control experiments with ischemic pig eyes have shown a strong decrease in light responses for longer ischemia times; see also [46]). Further, we recorded only from retinal pieces in the hemisphere opposite of the one containing the tumor.

**Fig 6:**
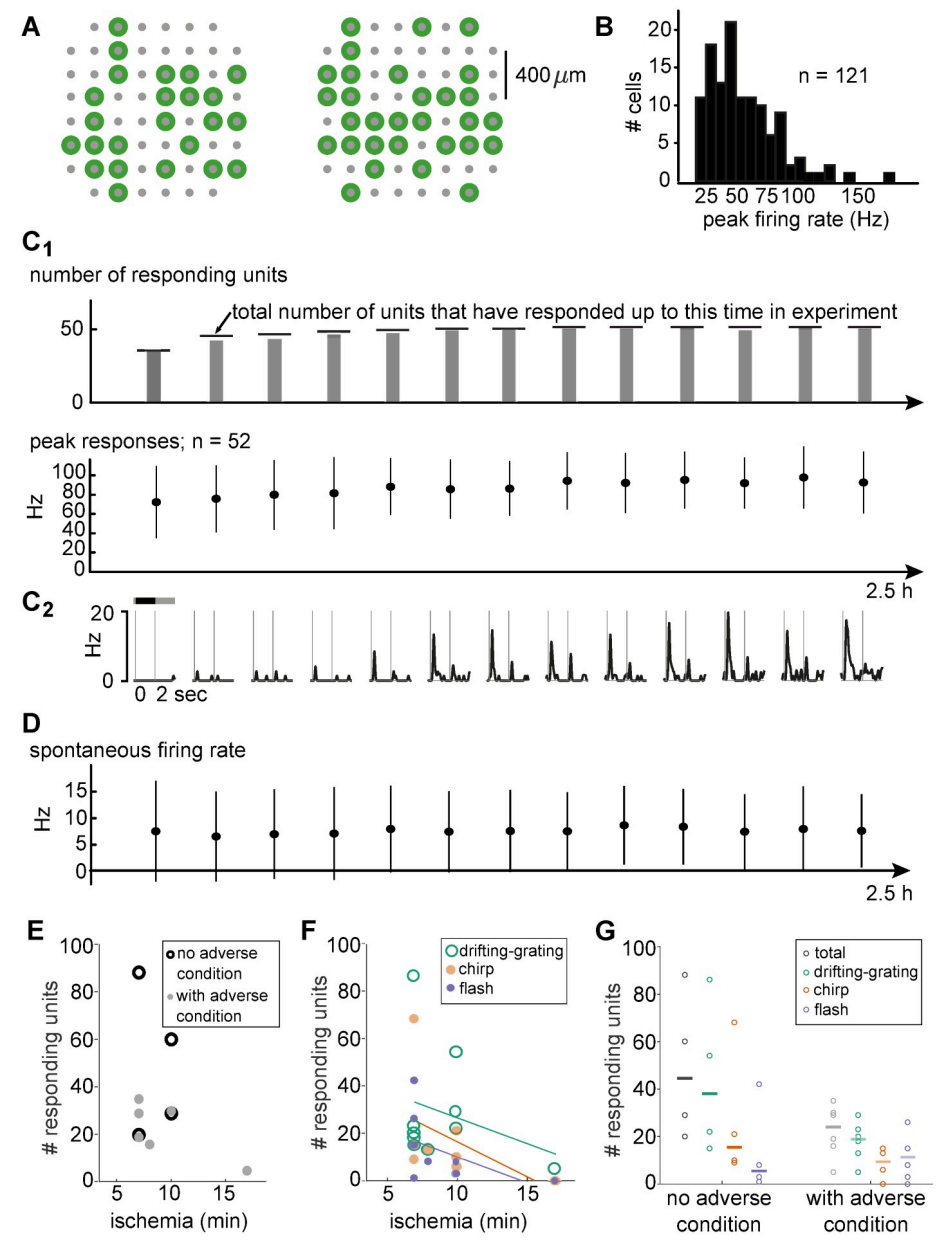
Donated human retinas are healthy. (A) Multi-electrode array layout (gray) and electrodes with sortable, light-responsive cells (green) of two example experiments. Responding cells are distributed across the recorded retinal pieces. (B) Distribution of peak firing rates in response to full-field contrast steps. (C_1_) Top: Number of cells responding at any given time point (gray bars) was similar to the total number that had responses until that time point (horizontal lines). Bottom: Mean ± standard deviation of peak firing rate for the responding cells. Data from a subset of experiments that lasted for 2.5h (N = 4 experiments, N = 52 cells). (C_2_) Example firing rate traces for one cell. (D) Spontaneous background firing rates (mean ± standard deviation) of the same cells and time points as in C_1_. (E) Number of responding units and ischemia time for donors without (black) and with (gray) known adverse condition (indicated in Table 1). (F) Number of responding cells for individual stimuli as a function of ischemia time. Lines indicate linear fits. (G) Total number of responding cells and responses per stimulus for donors with and without known adverse condition. Lines indicate median numbers.

We performed several tests to assess the health status of the donor tissue. First, light-responsive cells (displayed as green circles overlaid over the MEA electrode grid in Fig 6A) were distributed across the retinal piece, indicating good recording conditions. Overall response strength is another indication of tissue health. We compared the response strength of the recorded cells in the human retina with published primate data, and computed the firing rate in the same way as previous publications on macaque retina [47]. We then extracted the peak firing rate for each cell to the full-field contrast steps. Fig 6B shows the distribution of peak firing rates: many cells produced maximal responses of 20–90 Hz, but peaks could reach up to 180 Hz. Under comparable conditions (binary full-field noise), Uzzell & Chichilnisky report example cells with response peaks between <80 and 300 Hz [47]. The amplitude of the human response peaks reported here is hence in the same range as found in macaque retina.

Peak firing rates were not only comparable to published monkey data but were also stable throughout the experiments. Four retinal pieces were recorded for 2.5 hours and we computed peak responses averaged across blocks of 5 full-field contrast steps across the whole experiments. While some cells did not respond to this stimulus in the very beginning of the experiment, they responded consistently once they started and their peak firing rates were stable (Fig 6C_1_). The responses at each time point (averaged across blocks of 5 steps) for an example cell are shown in Fig 6C_2_. Similarly, spontaneous background spiking activity was stable across the full recording (Fig 6D). Taken together, the recorded human retinas did not show typical signs of deteriorating or degenerated tissue and exhibited stable spontaneous and evoked activity.

Finally, the ischemia duration experienced by our donated retinas were comparable to the times in other studies [19, 45]. In our data set, we found similar numbers of responsive units for ischemia times of 7 and 10 minutes (Fig 6E). The number of responsive cells decreased with increasing ischemic duration, consistent with the observation that we had no responding cells in three out of four retinas with ischemia times beyond 15 min (retinas 4, 6 and 8, see Table 1). When looking at the results in more detail, we could not detect a shift in responses to certain visual stimuli with increasing ischemia time, but rather there was a similar decrease in number of responsive units to all stimuli (Fig 6F). There appears to be some impact of the health status on the number of responsive cells: while ischemia times were comparable between retinas from otherwise healthy donors and donors with a relevant adverse condition (8.5 and 7.5 min on average), the retinas with very high numbers of responding units originated from donors without known adverse condition (Fig 6E and 6G). However, as with the ischemia time, we only observed a general decrease in the number of responsive units, not a shift that emphasized responses to certain visual stimuli (Fig 6G).

## Discussion

In this study, we describe light response properties of human retinal ganglion cells and find that these properties are very diverse. We found cells that responded only to positive contrast steps (ON cells), cells that responded only to negative contrast (OFF cells), and cells that encoded both positive and negative contrast (ON-OFF cells). The recorded human ganglion cells preferred different spatio-temporal stimulus frequencies and had distinct response properties when presented with local stimuli or contrast ramps. This diversity is consistent with the variety of cell types predicted by morphological classification in primate retina [11, 22]. Our extensive dataset of 324 light-responsive ganglion cells provides an overview of the visual features routed by the human retina to the brain and suggests a similar richness of information processing in the primate retina as found in other mammals where recent studies estimate over 40 distinct retinal information streams [2, 3] and which is consistent with reported anatomical [11] and molecular [12] diversity in human retinal ganglion cells.

### Putative midget, parasol and ON-OFF bistratified cells

Based on chirp and spatio-temporal response properties, we identified 16 clusters of retinal ganglion cells. We clustered responses of these retinal ganglion cells to a ‘chirp’ stimulus which has been previously used to group human retinal ganglion cell responses [45]. In 43% of our light-responsive cells, we found response properties that fit one of the five clusters in [45] and which putatively correspond to the five dominating primate retinal ganglion cells (ON and OFF parasol and midget cells, and ON-OFF bistratified cells (Fig 5A)). These data suggest that it is possible to record previously characterized response properties of non-human retinal ganglion cells also in *ex-vivo* human tissue (see also [19]).

We assigned putative midget and parasol cells based on their ‘chirp’ responses. In the resulting clusters we found a rather broad range of spatial and temporal preferences in response to drifting-grating stimuli (Fig 5A_4_). This is partially due to how we computed preference indices: When fitting a Gaussian to the heatmaps of drifting-grating responses, one might not be able to correctly identify peaks at the extremes of the tested temporal and spatial frequencies since there exists no data for neighboring frequency combinations. Further, especially for cells with a very broad response pattern like parasol and midget cells, the peak response may be recorded for any of the many stimulus combinations that trigger a strong response, leading to a broader distribution of peak spatial and temporal frequencies within a cluster. Additionally, varying eccentricity of our recordings might be another reason for the differences in stimulus preferences within clusters. Especially the spatial frequency preferences of cells will tend to increase with eccentricity due to their increasing size and decreasing cell density.

### Additional response properties in the human retina

Studies of primate retina often aim to characterize in detail a selected type of ganglion cell. In the present study, we considered all cells with light responses, and did not select for specific response features. This approach led to the characterization of response properties that have not been assigned previously to a primate ganglion cell type. In addition to the five dominant cell types, we found 3 clusters of cells that responded to the same chirp stimulus, but with a preference for low and medium temporal frequencies, which is different from the putative midget and parasol cells. Importantly, half of our cells did not respond to the ‘chirp’ stimulus but showed consistent responses to a set of drifting-grating stimuli. Based on these responses, we could group these cells into 9 clusters with distinct spatio-temporal preferences.

The lack of responses to full-field stimuli and the specific spatio-temporal preferences suggest that some of these cells are responsible for detection of local stimuli of different size and speed. Indeed, we found very narrow tuning in some of these clusters, for instance to specific temporal frequencies (Cluster 9 and 15) or spatial frequencies (Cluster 13). Other putative cell types appear to respond to almost any stimulus property as long as the stimulus is not spatially uniform and/or is moving (Cluster 14). We also found potential candidates for speed-encoding cell types, which is characterized by their diagonal heatmap patterns (Cluster 10 and 12) with responses to different combinations of spatial and temporal frequencies that result in the same stimulus speed. Further experiments will be necessary to assign those clusters to anatomically or molecularly identified cell types in the primate retina.

### Comparison to published human retina light responses

Our reported data set differs in some aspects from published anatomical and functional data: Two recent publications report an estimated 5 [45] and 9–10 [20] functional cell types in their recordings from human retina. These numbers are significantly lower than our reported 16 clusters. In addition, anatomical and physiological data suggests that 60–80% of the peripheral ganglion cells are midget and parasol cells [11, 19], while in our data set only 39% were identified as putative midget or parasol cells. Finally, in our data set around 50% of the cells responded to the ‘chirp’ stimulus while Cowan and colleagues report ~80% of chirp-responsive units [45]. We believe that there are three main reasons for these discrepancies.

First, in any study, there is a potential bias for certain cell types due to the recording and data processing technique. In this study, we have used a perforated MEA, which tends to improve the contact of the tissue with the recording electrodes, while the other studies have used smooth MEAs. Differences in tissue contact can change the relative chances with which certain cell types are recorded depending on their size and location of their cell bodies. Further, we highly curated our data set and excluded any contaminated units. The goal of Cowan et al. 2020 [45] was to demonstrate the health of the retinal tissue rather than a comprehensive classification of response properties and similar curation has not been done for their data set. Taken together, these technical differences might have led to experiment-specific biases for different cell types and mixing of responses from several cells in non-curated data sets.

Second, the selection of visual stimuli creates a strong bias of how easily different cell types can be detected. To pursue their particular goal, the study of Cowan and colleagues used only one type of visual stimulus, the ‘chirp’. Similarly, the other two studies using human retinas have chosen white-noise stimuli to test for particular properties of midget and parasol cells [19, 20]. It is known from studies in other species that cell types that are specialized for detection of local patterns, motion (direction) or orientation do not respond well to these types of stimuli [48, 49]. Thus, we believe that the particular stimulus choices in these studies have led to an underestimation of the number of cell types that comprise the 20–40% of non-midget/parasol cells. In our study, which did not pursue detection of a particular cell type and included a broader set of visual stimuli, we estimate that the human retina contains at least 16 different cell types (2 midget + 2 parasol + 12 others), which corresponds well to the number of ganglion cell types expected from anatomical (17 types) [11] and transcriptomic (18 types) [12] analysis.

Third, our data processing might have created a bias against midget and parasol cells. While we believe that the number of non-midget/parasol cell types has been underestimated in other studies, the evidence for a strong dominance of midget and parasol cell in terms of numbers is clear on anatomical and functional levels. We would hence have expected that at least 60% of our recorded cells would be assigned to the putative midget and parasol clusters. During the rigorous curation of our data sets, we excluded units that were not clearly separable and had too low inter-spike-intervals. Such mixing of units may happen if two cells with very similar waveforms and response timings are recorded by the same electrode. Due to the overrepresentation of midget and parasol cells, the chances are high that we recorded more than one cell of the same parasol/midget type on the same electrode and we hence might have excluded their spikes more often from analysis than for other cell types.

### Possibility of overestimation of diversity

Although our estimated number of cell types corresponds well with the expected number of cell types based on anatomy and molecular identity, the particular diversity of responses that we describe in this study might have been influenced by three main aspects. First, most of our stimuli were large and with spatial patterns in only one dimension, and thus responses likely reflect center-surround interactions. These interactions can be very diverse across different cell types, such that more global stimuli might help to distinguish cell responses that may otherwise be very similar during local stimulation. At the same time, the specific surround circuitry can depend strongly on the exact stimulus conditions including stimulus size [50] and absolute light level [50–52]. This dependency can contribute to an overestimation of the diversity of cell types across different recordings. In particular, the diverse history of the donor tissue (age, health and genetic background of the donor, see the third point below) may have consequences for the surround contribution such that full-field stimulation may exaggerate differing responses in cells of the same type across different recordings. However, under our controlled stimulus conditions, it is not very likely that our large stimuli caused much artificial response variability. What is more, we might even have under-estimated the diversity in the responses as we probably have not recorded from cells that only respond to very local stimuli.

Second, it is possible that some of the recorded cells are amacrine instead of ganglion cells. In addition, variety in the eccentricity of the retinal pieces may have introduced additional diversity in the response properties. This being said, non-human primate studies investigating specific ganglion cell types tend to focus on a large proportion of the retina (e.g. 25–70 degrees in [27], 30–60 degrees in [17]), but did not report any significant differences in response properties across eccentricities.

Third, we cannot exclude that the donor’s health status could have altered the responses of the ganglion cells. Still, on average, the human ganglion cells recorded here showed a similar response behavior to drifting-grating stimuli of different temporal and spatial frequencies as previously published non-human primate ganglion cells. While we found a trend towards fewer responding units in patients with known aversive conditions, the distribution of responses to different visual stimuli was similar as in patients without known adverse conditions. The most abundant cell types in the primate retina are midget and parasol cells. Our dataset contains cells that responded similarly to stimuli as previously described midget and parasol cells in other primates. Furthermore, the spatial threshold for the whole population of recorded cells (1.55 cyc/°) is comparable to psychophysically determined spatial resolution thresholds of human subjects measured at comparable eccentricity. This, together with the fact that we observed responding ganglion cells distributed across many recording electrodes, suggests that we were able to record physiologically relevant response properties in these donor human retinas.

### Absence of direction-selective cells

One of the best studied ganglion cells in non-primate mammalian retina are the direction-selective ganglion cells. It is unclear whether cells responding to a specific direction of movement exist in the primate retina. So far, no physiological recordings of direction-selective cells have been published (but see [53]) and we could not detect such direction-selective behavior in our data set either. Morphological studies identified potential candidates for primate direction-selective neurons [24, 54, 55]. These cells have a large dendritic field and hence they are much fewer in number than the smaller midget or parasol cells. Consequently, the chances to record from such large cells in unbiased MEA experiments is small. Furthermore, as shown in the present study as well as in previous measurements [56], primate ganglion cells respond to higher temporal frequencies than for instance mouse ganglion cells [57]. It is therefore possible that our and other studies missed direction-selective cells in primate retinas due to suboptimal stimulation paradigms. This should be taken into consideration for future studies.

### Future studies on the output of the human retina

In this study we provide the first non-selective description of the retinal output in humans. We showed that our data is consistent with measurements in other primates and that the diversity in the human retinal output is larger than suggested by previous physiological studies that focused on only a few primate retinal cell types. To further investigate these unstudied ganglion cells and to achieve classification into individual cell types, one could make use of high-density MEAs. These MEAs allow recording from almost every cell in a given patch [33], and local stimulation would be possible to test for parameters such as center-surround mechanisms, local edge detection or approach sensitivity. It has been shown that each ganglion cell type tiles the retina with little overlap in order to encode every visual feature at each point in the visual field [58]. Such mosaic formation can as well be revealed with high-density MEA recordings [16, 17, 19, 20, 25] and can then be used for cell type identification.

### Impact on bio-medical research

The goal of bio-medical research is to better understand human physiology and to find treatments in the case of disease. Knowledge about the detailed functioning of the human retina would be desirable also in the context of retinal diseases. Such diseases, in particular blindness, have a big impact on individuals and the society. In recent years, research has yielded some promising approaches to potentially healing blindness (e.g. electrical retinal implants [59, 60], optogenetics [61, 62], stem cell therapy [63]) with a common ultimate goal: to come as close as possible to full vision capabilities by interfering appropriately with the retina of the patient. Especially optogenes (light sensitive ion channels/pumps) are a promising tool to render degenerated photoreceptors, bipolar cells, or ganglion cells light sensitive [61, 62, 64–66]. Currently, these treatment options are mostly developed and tested in animal models. We see a big advantage of supplementing this research with human retina studies. First, increased knowledge about signal processing within the human retina may support further and faster progress in that field. Second, cell type specificity of viral vectors and the correct expression of the genetic construct containing the optogenes could be developed using *ex-vivo* or *post-mortem* human retina. Moreover, by subsequent comparison of the optogene-driven light responses with the natural responses presented in this and future studies, one could evaluate the efficacy of the treatment. Finally, (side-)effects of drugs such as neuroprotectiva (substances to conserve as much as possible of leftover visual capabilities) could be tested directly on human retina instead of using porcine, bovine or other animal models. First studies have started using human tissue to characterize the genetic profile of human retinal cells and to test first treatment options [67, 68]. We hope that the present study may serve as encouragement for more research with *ex-vivo* human retina in the future.

## Methods

Code to recreate several figures and the necessary spike times and processed data can be accessed on GitHub (https://github.com/katjaReinhard/HumRet) and spike times as well as processed data on OSF (https://osf.io/zf9rd/).

### Human retina donations

To characterize information processing in the retina, very fresh tissue is necessary because the photoreceptors rapidly lose light-sensitivity. We obtained such human retina from patients of the University Eye Hospital in Tübingen, who had to undergo enucleation of one eye, usually to remove a tumor. The authors were not involved in recruiting donors or receiving consent. All participants provided informed, written and verbal consent to their ophthalmologist to the use of their retina for scientific research purposes after the eye had been removed. No tissue in addition to the medically necessary procedure was removed for this study and all donations were collected in a pseudonymous fashion (patient data is kept at the University Hospital, authors only received a number and non-identifying data such as age, sex, and pre-existing conditions). All procedures were approved by the ethics committee of the University of Tübingen (approval number 531/2011) and performed in accordance with the guidelines and regulations provided by the ethics committee.

The retina was protected from light during surgery if possible. An ischemia time of at least five minutes during the surgery (clamping of the optic nerve before removing the bulbus) was mandatory to prevent strong bleeding. The bulbus was cut in halves directly after enucleation, and the hemisphere without tumor was put immediately into CO_2_-independent culture medium (Gibco, ThermoFisher Scientific, Massachusetts, USA), kept in darkness at room temperature and transported to our lab. Under dim red light, we removed the vitreous and cut small mid-peripheral retinal pieces (~ 3x3 mm^2^). Within 23 months we obtained 15 such ex-vivo donations (Table 1). 15 pieces from 10 retinas were used for experiments.

### Experimental design

To maximize the amount of information gained from the rare experiments with fresh human retina, we employed recordings with flat multi-electrode arrays (MEA) that allow for measuring the activity of many neurons in parallel [38]. MEAs include a square or rectangular electrode arrangement that is brought in contact with the ganglion cells, allowing measuring the retinal output in response to light stimulation. Our MEA experiments have been described in detail elsewhere [69]. Briefly, the retinal pieces were placed ganglion cell side-down on a MEA. We used perforated 60-electrode MEAs with 200 μm distance between the electrodes (60pMEA200/30iR-Ti-gr, Multichannel Systems, Reutlingen, Germany). Then, various light stimuli were focused onto the photoreceptors with a Digital Light Processing projector (Sharp PG-F212X-L, Sharp Corporation, Osaka, Japan or Acer K11, Acer, Taipeh, Taiwan), and we recorded the output of the retina (i.e. the action potentials of ganglion cells in response to the stimuli) at 25 kHz with a USB-MEA-system (USB-MEA1060, Multichannel Systems) or an MC-Card based MEA-system (MEA1060, Multichannel Systems). During the experiments, the retina was kept at 25°C and continuously superfused with Ringer solution (in mM: 110 NaCl, 2.5 KCl, 1 CaCl_2_, 1.6 MgCl_2_, 10 D-Glucose, and 22 NaHCO_3_; ~270 mosm) or modified Ringer solution (in mM: 115 NaCl, 2.5 KCl, 2 CaCl_2_, 1 MgCl_2_, 15 D-Glucose, 1.3 NaH_2_PO_4_*H_2_O, 0.5 L-Glutamine, and 25 NaHCO_3_; ~285 mosm), both equilibrated with carbogen (95% O_2_, 5% CO_2_). All experiments were conducted with the retinal pigment epithelium removed.

### Light stimulation

The stimulation intensity provided by our projectors spanned 3 log units of brightness between a black (‘0’) and white (‘255’) stimulus. The projector output was linearized, so that the grey (‘128’) background was midway between black and white, and the intensity step between black and grey and between grey and white had equal amplitude. Recordings were performed at photopic intensity levels (light intensity for day vision) with a mean illuminance of 8·10^4^ rod isomerizations per rod per second. In two retinal pieces, no clear responses could be detected at this light level, and we used data obtained at a mean illuminance of 8·10^5^ rod isomerizations per rod per second for analysis. Note that recordings at photopic light levels do not necessarily imply that the observed light responses were driven by cones alone, rods may have contributed as well [70]. A broad set of light stimuli was used; each stimulus was repeated several times during recording sessions of two to six hours. We calculated various parameters from the ganglion cells’ responses (see below). To convert stimulus sizes on the retina (in μm) to the equivalent visual angles (in degree), we used the conversion factor 266 μm/° [71]. We discuss in this article six parameters extracted from responses to the following six stimuli (see also Fig 1):

#### Sinusoidal drifting-gratings

Drifting sinusoidal grating stimuli with 24 different combinations of spatial periods and temporal frequencies (1, 2, 4, 8 Hz; 100, 200, 500, 1000, 2000, 4000 μm spatial period on the retina) were used for spatio-temporal analysis (Fig 1A). The gratings were shown at full contrast (‘0’ to ‘255’) and moved in one direction for 12 seconds.

#### Temporal and contrast chirp

Temporal tuning was also tested with a spatially homogeneous chirp stimulus [2], i.e. full-field frequency-modulated intensity change between black (‘0) and white (‘255’), according to: *intensity* = 128+128*sin(*π*(*t*^2^+*t*/10)), with t given in seconds. The temporal frequency increased from 0.5 to 8 Hz over a time course of approximately 8 seconds (Fig 1C top). In addition, a contrast ramp increasing from none to full contrast within 8 seconds was used to test for contrast sensitivity (Fig 1C bottom).

#### Single bars at various velocities

We used single bars moving with different speed to test for speed preferences. A bar with 1000 μm extension in the movement direction (either black or white) and covering the complete screen in the other direction moved in front of a gray background in one direction (same direction as grating stimulus) with different speeds (1, 2, 4, 8, 16 mm/s) with a gap of 3 seconds before the next higher speed (Fig 1E).

#### Full-field contrast steps

Full-field contrast steps were applied for measurements of response polarity and latency (Fig 1G). A single stimulus consisted of four transitions (grey → black → grey → white → grey) spanning the full projector intensity of 3 log units of brightness (contrast for each step: ± 1 Weber contrast). Each contrast step lasted for 2 seconds.

#### Direction-selectivity

We used a single bar (black or white) moving in 8 directions to test for direction-selectivity. The bar of 1000 μm width was moved with 1 mm/s across the retina.

### Spike extraction

Spike sorting (assignment of single action potentials to individual cells) was performed with an in-house MATLAB (MathWorks, Massachusetts, USA) routine written by Alexandra Kling. Different features of the action potential waveforms, such as amplitude, width, or principal components, were calculated and projected onto 2-dimensional space to separate action potentials of different cells from each other and from noise. In addition, the spike refractory time of all spikes of a sorted cell had to be >1.5 ms. After spike sorting, we determined light-responding cells by visual inspection of the activity to all stimuli. To calculate the firing rate, the spike train was convolved with a Gaussian and plotted against time. The sigma of the Gaussian varied for different analysis purposes; the value applied in each case is given in the description below. For the firing rates of the example cells in Fig 2, σ = 40 ms was used. Only cells for which spikes could be sorted confidently were used for analysis (for consistency, the same person performed spike sorting for all experiments and applied the identical quality judgement system). We applied cross-correlation analysis to detect recordings from the same cell on different electrodes (e.g. from cell body and axon). In this case, only one of the recorded units was used for the analysis.

### Response parameter calculation

#### Spatio-temporal tuning

Drifting sinusoidal grating stimuli were used for spatio-temporal analysis. First, cells responding to at least one of the drifting gratings were identified manually. For each cell and stimulus repetition we represented the cell’s activity with a binary vector indicating the presence (1) or absence (0) of a spike (time bins: 1 ms). For each drifting grating stimulus, we then calculated the mean of these binary spike rates across repetitions of the same stimulus, and computed its Fourier transform (FT). The FT peak at the stimulus frequency was then taken as the cell’s response strength. The Fourier transform was considered to have a peak (i.e., the cell was considered to respond to the stimulus) if there was no higher peak at any other frequencies (excluding multiples of the stimulus frequency).

#### Temporal tuning

Temporal tuning was tested with a chirp stimulus, i.e. frequency-modulated sinusoidal full-field change of intensity. We calculated the FT of both, the stimulus and the response (mean binary spike train, frequency resolution of 0.125 Hz). Response strength along the stimulation frequencies was defined as *norm*(*FT_response_*)/*FT_stimulus_*. Fluctuations were smoothed; these appeared especially at low temporal frequencies due to the timing of ON- and OFF-responses. Smoothing was achieved by averaging of the response strength with a moving average across a 3-datapoint-window (0.375 Hz) in steps of 1 data-point (0.125 Hz). Population data is presented in 2.6 Hz bins across all responding cells (Fig 1D).

#### Median speed preference

A black or white bar was moved across the retina in one direction (same direction as drifting granting) with various speeds. The cumulative sum of peak responses for each speed (firing rate calculated with σ = 40 ms) was computed. The speed value for which 50% of the cumulative sum was reached was taken as the cells’ median speed preference. For each cell that responded to both, white and black bars, the higher preferred speed was taken for the population plot in Fig 1F.

#### Polarity

Polarity was defined based on the responses to full-field contrast steps. Cells with responses only for positive contrast steps were considered as ON-cells; OFF-cells had detectable responses only to negative contrasts, and ON-OFF-cells responded to both types of contrast steps. Firing rates were calculated by convolving the spike rates with a Gaussian (σ = 40 ms). The cell was considered to show a response if the peak firing rate was bigger than mean spontaneous activity + 2 standard deviations (measured before the first contrast step).

#### Transiency

Response transiency was defined as the time between the peak response to full-field contrast steps and returning of the spiking activity to baseline firing rate + 2 standard deviations. Shorter times correspond to more transient responses.

#### Gaussian fit to spatio-temporal heat-maps

We fit a Gaussian to the spatio-temporal response heatmaps to calculate frequency preferences and specificity of the response. The underlying code can be found on GitHub (https://github.com/katjaReinhard/HumRet). The center point and hence the preferred overall spatial and temporal frequencies were defined as the dot product of the heatmap with a mesh of spatial and temporal frequencies, respectively. The dot product of the squared distance of each heatmap point (SF for spatial, TF for temporal frequency) with the Gaussian center (Csf, Ctf) and the heatmap itself (peaks) was then calculated (*a* = (*SF*−*Csf*)^2^⋅*peaks, b* =((*TF*−*Ctf*)*(*SP*−*Csf*))⋅*peaks, c* = (*TF*−*Ctf*)^2^⋅*peaks*). The squared Eigenvalues of [a,b;b,c] were considered the major axes of the Gaussian.

#### Contrast preference index

To compute the contrast preference index, we took the background-subtracted response to the contrast part of the chirp stimulus and considered the first third (low contrast) and last third (high contrast). The index was calculated as *CPI* = (*high−low*)/(*high+low*) with ‘high’ indicating the sum of the absolute firing rate during high contrast and ‘low’ the sum of the absolute firing rate during low contrast. Cells that prefer low contrast have an index of <0, high-contrast preferring cells an index of >0.

#### Chirp frequency index

The chirp frequency index was based on the Fourier transforms of the responses to the frequency part of the chirp stimulus. We used the ‘fft’ function in MATLAB to calculate the fast Fourier transform (FFT) of each cell’s response as well as of the stimulus. We then divided the FFT of the response by the FFT of the stimulus and smoothed the resulting FFT ratio with a moving average across 0.3 Hz windows. The index compared the FFT ratio for high (6–7.2 Hz) and low frequencies (0.7–3 Hz) and was calculated as *CFI* = (*high−low*)/(*high+low*). The index indicates the preference of the cell for higher (>0) or lower (<0) temporal frequencies.

### Clustering of human retinal ganglion cell responses

Clustering of the light-responsive cells followed a 5-step process (Fig 4): 1) Assignment of cells to the 5 clusters in Cowan et al. 2020 [45] based on their responses to flash and chirp stimuli. 2) Assignment of cells with chirp, but without flash responses, to the 5 Cowan clusters. 3) Clustering of remaining chirp-responding cells (that did not fit into the Cowan clusters) based on their flash response. 4) Clustering of remaining cells based on their drifting-grating response. 5) Assignment of cells that were not probed with the chirp stimulus to the clusters established in steps 1–4.

#### 1) Assignment of cells to the 5 Cowan clusters (Cluster 1–5)

In Cowan et al (2020), the authors presented the same chirp and flash stimuli to human ganglion cells as we did. In their clustering, they identified 5 main clusters. We used the response profiles of those clusters as templates. We obtained the normalized, average response traces for the 5 clusters in Cowan et al. 2020 Fig 4 from the authors. Each cell with chirp and full-field flash responses was assigned to one of the 5 Cowan clusters based on the following criteria: polarity (identified as described above), transiency (as described above) high vs low frequency response (median response for high temporal frequencies vs median response for low temporal frequencies of the chirp stimulus). Cells were assigned as follows:

transient ON cluster (putative ON parasol): ON polarity, transiency < 400 ms, high > low
transient OFF cluster (putative OFF parasol): OFF polarity, transiency < 400 ms, high > low
sustained ON cluster (putative ON midget): ON polarity, transiency ≥ 400 ms, (high-low)<0.2
sustained OFF cluster (putative OFF midget): OFF polarity, transiency ≥ 400 ms
ON-OFF cluster (put. ON-OFF bistratified): ON-OFF polarity, transiency < 400 ms, high > low

#### 2) Assignment of cells with chirp, but without flash responses (Cluster 1–5)

We measured for every cell with chirp responses its similarity to all 5 Cowan clusters. Similarity was defined for each cell x and Cowan cluster template c as *Similarity_xc_* = ∑|(*normResponse_x_*−*template_c_*|, resulting in 5 values for each cell. Further, we calculated the average similarity of cells assigned in step 1 for each of the 5 clusters (*mean_c_*). Cells that could not be assigned in step 1 because they did not respond to the full-field flashes were now assigned to the 5 Cowan clusters if *Similarity_xc_*≤*mean_c_+std_c_*.

#### 3) Clustering of remaining chirp-responding cells based on their flash response (Cluster 6–8)

After step 1 and 2, we were left with cells that did respond to the chirp, but in a different way than the 5 Cowan clusters, and cells that did not respond to the chirp stimulus. We grouped the former into three additional clusters based on their flash response. We did not find any remaining cells with ON-OFF responses, hence, the resulting clusters were ON-cells with chirp responses, OFF-cells with chirp responses, and cells with chirp but without flash responses.

#### 4) Clustering of remaining cells based on their drifting-grating response (Cluster 9–16)

Cells in cluster 1–8 are defined by the fact that they do respond to chirp stimuli. We found that around 50% of our cells did not respond to the chirp stimulus and hence could not be grouped into cluster 1–8. Most of these cells, however, responded well to drifting-gratings and were grouped based on these responses. The 24 peak responses were normalized for each cell so that for each cell, the response to its optimal drifting-grating was 1. These normalized responses were clustered using k-means in MATLAB (squared Euclidean distance, 1000 repetitions). The best number of clusters was identified using the Calinski-Harabasz [72] and Davies-Bouldin indices [73] (‘evalclusters’ in MATLAB).

#### 5) Assignment of cells that were not probed with the chirp stimulus

Finally, our data set contained cells that were not probed with a chirp stimulus. We could hence not cluster those cells according to our 5-step process, but we could estimate their best fit based on their drifting-grating responses. Similar to step 2, we calculated the similarity of each remaining cell with the average 24 drifting-grating responses of Cluster 1–16. Cells were assigned to the cluster with the smallest similarity value. Cells that did not fall within the 95% of the already present similarity values within a cluster were not assigned to any cluster.
